# Identification of Object Dynamics Using Hand Worn Motion and Force Sensors

**DOI:** 10.3390/s16122005

**Published:** 2016-11-26

**Authors:** Henk G. Kortier, H. Martin Schepers, Peter H. Veltink

**Affiliations:** 1 Institute for Biomedical Technology and Technical Medicine (MIRA), University of Twente, P.O. Box 217, 7500 AE Enschede, The Netherlands; p.h.veltink@utwente.nl; 2 Xsens, Pantheon 6-A, 7521 PR Enschede, The Netherlands; h.m.schepers@xsens.com

**Keywords:** inertial sensors, force sensors, on-body measurements

## Abstract

Emerging microelectromechanical system (MEMS)-based sensors become much more applicable for on-body measurement purposes lately. Especially, the development of a finger tip-sized tri-axial force sensor gives the opportunity to measure interaction forces between the human hand and environmental objects. We have developed a new prototype device that allows simultaneous 3D force and movement measurements at the finger and thumb tips. The combination of interaction forces and movements makes it possible to identify the dynamical characteristics of the object being handled by the hand. With this device attached to the hand, a subject manipulated mass and spring objects under varying conditions. We were able to identify and estimate the weight of two physical mass objects (0.44 kg: 29.3%±18.9% and 0.28 kg: 19.7%±10.6%) and the spring constant of a physical spring object (16.3%±12.6%). The system is a first attempt to quantify the interactions of the hand with the environment and has many potential applications in rehabilitation, ergonomics and sports.

## 1. Introduction

A prosperous recovery of arm and hand function after a neuromuscular accident requires adequate training of reach and grasp tasks. It is in many applications desired to evaluate task performances in a quantitative manner. However, the usual procedure is that patients visit the rehabilitation center on a regular basis where either the physical therapist (PT) or rehabilitation specialist (RS) assesses the patient’s performance using subjective measures. Hence, such an evaluation is just a snapshot of the patient’s capabilities under specific physical and environmental conditions. A continuous monitoring system that informs the PT with quantitative measures obtained in activities of daily living (ADL) would provide more detailed information about the patient’s recovery.

A full evaluation of motor task performance during grasp movements requires, besides kinematic, also kinetic measures. Gathering both kinematic and kinetic measures allows for a full dynamic evaluation of tasks in terms of, e.g., movement trajectory, exerted forces, power exchange and the identification of load and body dynamics. In fact, such measures give much more detailed information of the subject’s performance compared to solely movement registration and, thus, allow for more specific clinical outcomes.

Previously, we demonstrated the principle feasibility of estimating power exchange by handling passive spring and mass loads [1]. Subsequently, we were able to identify and, moreover, estimate the load characteristics of a system by manipulating its position [2]. However, the instrumentation being used was rather bulky and, therefore, not suitable for on-body attachment.

Measuring interaction forces, tactile information or having a haptic perception is of interest in many domains [3]. Haptic robots, like DaVinci (Intuitive Surgical, Inc., Sunnyvale, CA, USA) or HapticMaster (Moog Inc., Buffalo, NY, USA), are able to accurately render any kind of contact dynamics, which allow, e.g., surgeons to perform remote operations under minimally-invasive conditions [4]. Those haptic devices require an accurate measure of interaction forces, which are preferably picked up as close as possible to the man-machine interface.

Tactile sensing is important when visual or auditory information is not available or disrupted. Hence, for both humans and robots, tactile information is valuable for identifying and planning strategies during the manipulation of loads [5,6].

Measuring interaction kinematics and kinetics is essential in the field of neuromuscular modeling [7,8] and estimation of muscular skeletal loading [9]. Identification of the neuromuscular system in interaction with the environment is in essence a closed-loop identification problem. This imposes several difficulties as most algorithms would estimate the lumped (environment and human body) dynamics. Separation requires known force or movement perturbation signals, such that models can be deduced that represent both active and passive body dynamics, as well as the environmental dynamics. However, it is difficult to simulate situations that mimic ADL because the human body is able to adapt its internal dynamics for environmental changes easily.

Various groups exploited different force sensing modalities. An in-depth overview and comparison is given by Youssef et al. [10]. They concluded that resistive sensors are still favorable compared to other modalities. However, in the last couple of years, new advances were made for a sensor that could be attached to the skin, like strain gauges [11], elastomers [12] or capacitive layers (FingerTPS, PPS Inc., Glasgow, UK). A recent review article of Almassri et al. [13] indicated that there is a transition from MEMS kind of solutions towards polymer and piezoresistive solutions in order to improve the haptic sensation. These sensing modalities have high potential, though it is still a major concern to discriminate between different sensing directions. Kurillo et al. [14] developed an isometric force measuring system to quantify grip forces of two fingers and the thumb. Their system used a model to transform the measured tip forces to a total wrench applied to a virtual object. Corresponding movements were subsequently determined by numerical integration. A multi-directional sensitivity is advantageous as it enables one to distinguish between different force components, which are attributed to the total measured force during the grasp of an object. To the authors’ knowledge, there is no commercially available sensor that allows measurements of interaction forces in multiple directions and that is suitable for placement on small surfaces, like finger tips.

Recently, Brookhuis et al. [15,16,17,18] developed a novel miniaturized six-axis microelectromechanical (MEMS) force/torque (FT) sensor. This sensor is specifically designed for biomedical purposes, such as the measurement of contact forces.

Kinematic estimates can be obtained using widely-available MEMS-based inertial and magnetic sensors. By applying a suitable sensor fusion algorithm, they allow an accurate estimate of the sensors’ orientation with respect to a global coordinate frame [19,20,21]. Subsequently, by applying forward kinematics, an estimate of the finger and thumb tip positions, velocities and accelerations are obtained, which have been demonstrated in our previous article [22]. More recently, this kinematic hardware (PowerGlove) was used in clinical tests for the assessment of patients suffering from Parkinson’s disease and monitoring the hand functioning of aging people [23].

The contribution of this paper is two-fold. First, an experimental system capable of measuring 3D hand and finger kinetics and kinematics is presented. The system combines a small, commercially available, inertial/magnetic sensor with a novel 6D force torque sensor, as described in the previous paragraph. The sensors are attached to the human hand and allow the measurement of interaction forces and movements simultaneously; Second, a method is presented to estimate the dynamic characteristic of a load being manipulated by the user’s hand. The method uses a recursive parametric approach, which is able to identify various properties of dynamic loads under time-varying conditions.

## 2. Method

**Figure 1:**
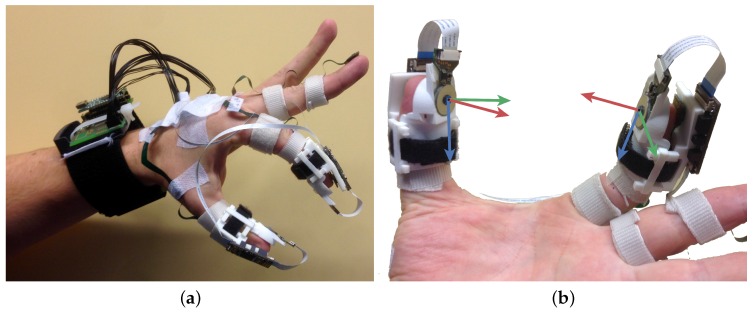
Force sensor instrumentation attached to the hand, index finger and thumb. The inertial kinematic instrumentation (described previously in Kortier et al. [22]) is attached to the dorsal side of the hand, index finger and thumb (**a**). Flexible PCBs allow the connection between the rigid sensor PCBs, which are placed on every digit. Custom-made cuffs (white 3D printed material) secure the force sensor attached to the tips and enforces a rigid connection to the local inertial sensor (**b**). Applied forces are transferred to the MEMS 6D force/torque sensor via a cylindrical (height = 3 mm, ⊘=4 mm) glass stylus with rubber coating (blue, origin of the coordinate frames). The force sensor coordinate system is visualized with the x-axis (blue), y-axis (green) and z-axis (red).

The instrumentation setup comprises 3D accelerometers and gyroscopes embodied in a single chip (ST LSM330DLC), which are distributed along the dorsal side of the hand. Onto all digits of the index finger and thumb, a single chip was attached. In addition, custom-made cuffs (3D printed plastic material) are attached to tips of the index finger and the thumb. Each cuff holds a 6D force/torque sensor on the palmar side of the tip; see Figure 1.

The force/torque sensor was developed and built by Brookhuis et al. [18]. Two silicon layers, suspended with a spring-like construction with a known stiffness, can be displaced with respect to each other. Any displacement results in a change of capacity, which gives a direct measure of the forces applied. Using a differential measurement approach, which is realized by comb structures, one can discriminate between various sensing directions. Eventually, capacity change is transformed via a modified martin oscillator to voltages, which are further processed by an Atmel ATmega16 microcontroller.

The custom-made cuffs enforce a rigid connection between the dorsal (inertial sensor) and palmar (force sensor) side of the tip. Hence, the relative orientation between the inertial and force sensor placed on the same tip is known at all times.

The inertial sensors are used to estimate both the global orientation of the hand and the hand’s internal pose (relative orientations of the phalanges). These orientation estimates are used to express all measured signals in a common reference frame (coordinate transformation). In addition, the inertial sensor outputs are used to estimate (relative) translational movements of finger and thumb tips by applying forward kinematics.

Kinetic outputs are used to measure the interaction forces between fingers and the object and to detect the contact between the finger and thumb with an arbitrary object.

Eventually, both kinematic and kinetic estimates are used as an input of the identification algorithm to estimate the dynamic characteristics of the objects manipulated by the hand.

In the next subsections, system identification methods (Section 2.1) and the methods for estimating all relevant kinematic and kinetic variables (Section 2.2) will be outlined.

### 2.1. System Identification

It should be noted that the relation between interaction force and movements at the interface of two bodies is, in general, determined by the dynamic characteristics of both bodies. Identification of such a system is a typical closed loop identification problem [2,24]. However, if the following conditions are met (a mathematical derivation can be found in Appendix A), forces applied by the human body divided by the common velocity yield an approximation of the load’s impedance:

1. The human body is an active generator of force:
  (a) The force generated by the human body and applied to an environmental object is minimally influenced by the joint movement of both bodies and can, therefore, be considered as an independent input.
  (b) The force generated by the human body and applied to an environmental object has a sufficiently high bandwidth, that is a larger bandwidth than the load.
2. The load is passive with a relatively low bandwidth.

These conditions are often satisfied in our daily-life interactions with the environment. We will demonstrate that identification of the load characteristics is indeed possible under the above conditions.

**Figure 2:**
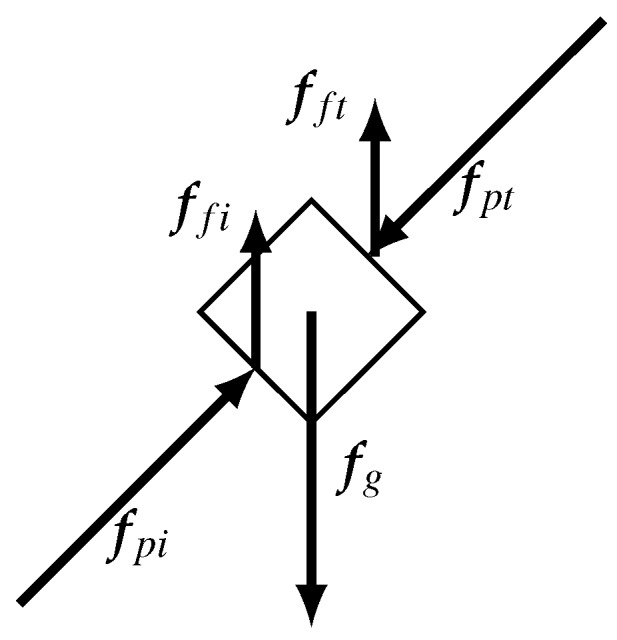
Force diagram of an stationary unknown load configuration grasped by the index finger and thumb. The index finger and thumb apply a pinching force indicated by $f_{p\{i,t\}}$. Friction $f_{f\{i,t\}}$ compensates for the gravity $f_{g}$ force acting on the load’s mass.

Consider a load with unknown internal dynamics being pinched by the index finger and thumb; see Figure 2. According Newton’s second law, the sum of forces acting on the mass when the object is pinched between index and thumb is given by:

(1) $$\sum f=f_{p}+f_{f}+f_{g}=ma$$

where $f_{p}$ is the pinching force, $f_{f}$ is the friction force, $f_{g}$ the gravitational force, *m* is the mass and a is the acceleration of the mass.

In general, the load can be manipulated in two different ways:

1. Inertial and gravitational perturbations act on the load, referred to as external dynamics; for instance, gravitational force (low bandwidth) or inertial displacements introduced by the arm. In addition, viscous and or elastic elements might be present. A non-zero net force would result in an acceleration of the whole object.
2. Internal perturbations of the dynamics; for instance, grasping or pinching, referred to as internal dynamics. The difference in pinch forces is related to the load dynamics according to Newton’s third law. The object exerts equal, but opposite forces.

**Figure 3:**
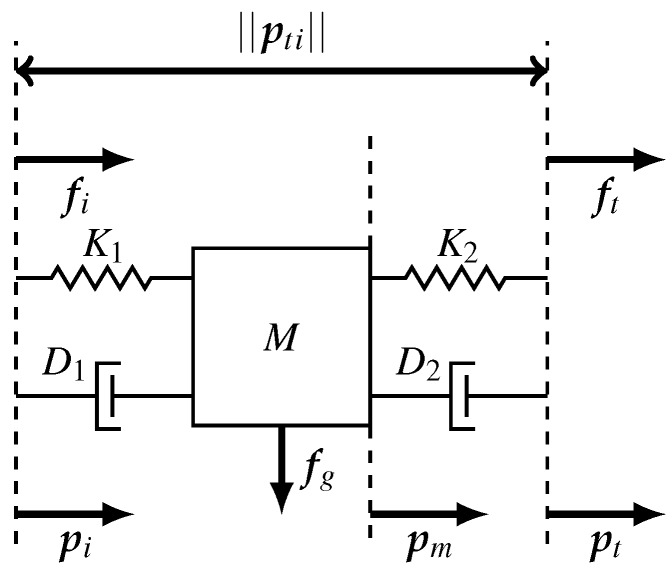
Model of the load configuration grasped by the index finger and thumb. A mass (*M*) is suspended by two spring ($K_{\left\{1,2\right\}}$) and damper ($D_{\left\{1,2\right\}}$) pairs. The index finger and thumb apply forces indicated by $f_{i}$ and $f_{t}$. The positions of the mass, index finger and thumb are indicated by $p_{m}$, $p_{i}$ and $p_{t}$, respectively.

In order to demonstrate the principle of load identification, rather simple environmental loads will be used that can be manipulated by the index finger and thumb. These loads can be modeled as a mass, which is suspended by a spring-damper pair; see Figure 3.

$f_{i}$ and $f_{t}$ are the measured forces at the contact interface of index finger and thumb, respectively. It is assumed that both springs ($K_{1}$ and $K_{2}$) and dampers ($D_{1}$ and $D_{2}$) have equal characteristics and are only defined in one direction, corresponding to the shortest vector between the application points of both tips. We assume that the internal dynamics obey a large bandwidth compared to the movement bandwidth. That is, either the stiffness is assumed infinitely large or the mass’ inertia is assumed to be negligible. Therefore, it is allowed to assume that the distance of the mass is approximately equal to both tips.

(2) $${\left|\left|p_{t}-p_{m}\right|\right|}_{2}\approx {\left|\left|p_{m}-p_{i}\right|\right|}_{2}$$

The total force exerted by springs and dampers is summed and modeled in a simple linear fashion:

(3) $$\begin{array}{cc} f_{s} & =-K\left(p_{ti}-p_{0}\right) \end{array}$$

(4) $$\begin{array}{cc} f_{d} & =-D{\dot{p}}_{ti} \end{array}$$

Now, for the first situation, i.e., when the (mass)load is accelerated, we assume that the relative acceleration of the mass with respect to the tips is negligible compared to the gravitational acceleration; see Equation (2) (${\ddot{p}}_{tm}={\ddot{p}}_{mi}\approx 0$); the summed forces can be rewritten as:

(5) $$f_{i}^{g}+f_{t}^{g}=f_{tot}^{g}=M\left(g^{g}+a^{g}\right)$$

where g and a are the gravitational and inertial accelerations, respectively. The superscript *g* implies that the vector is expressed in the global inertial reference frame.

The second situation, i.e., internal force perturbations, requires the difference in tip forces, which can be expressed as:

(6) $$f_{i}-f_{t}=f_{diff}=D{\dot{p}}_{ti}+K\left(p_{ti}-p_{0}\right).$$

Equations (5) and (6) can be written in a linear fashion for each time instance *k*:

(7) $$y_{k}=H_{k}\theta +e,$$

where $H_{k}$ is the measurement matrix, $\theta ={\left[\begin{array}{ccc} M & D & K \end{array}\right]}^{T}$ the parameter vector and e is an independent error term. Subsequently, Equation (7) can be stated as a linear optimization problem:

(8) $$\hat{\theta }=\underset{\theta }{arg min}\sum_{k=0}^{N}{\left(y_{k}-H_{k}\theta \right)}^{T}R_{k}^{T}\left(y_{k}-H_{k}\theta \right)$$

where we seek for the optimal parameter vector $\hat{\theta }$, such that the above function is minimized. Equation (8) can be solved easily using a weighted least squares solver. The covariance *R* matrix defines the uncertainty of different measured signals. Alternatively, Equation (8) can be written as a batched form, such that the parameter values are found recursively. This is advantageous because not all input data have to be collected prior to the start of the system identification process, and changes of the parameter values, which often occur in ADL tasks, can be estimated over time. A popular and robust algorithm to solve for varying parameters values is the recursive least squares (RLS) solution [25] and has therefore been applied.

### 2.2. Kinematics and Kinetics

As mentioned in the introduction of the Methods section, the kinematics estimates are necessary for two purposes.

1. Expressing all 3D sensor signals in the same coordinate frame. This allows further processing during the system identification step. This requires the relative orientation between different segments and the global frame.
2. Having a kinematic estimate of both the finger and thumb tip.

An extended Kalman filter (EKF) approach was deployed to estimate the optimal orientation between the sensor frame ($\Psi_{b}$) and global frame ($\Psi_{g}$) being expressed by an unit quaternion (${\hat{q}}^{gb}$). Such an approach has a good reputation as a sensor fusion strategy in terms of accuracy and computational load [26]. It uses the 3D gyroscope ($y^{g}$) as the primary input for the one step ahead predictor of the orientation:

(9) $$q_{k+1}^{gb}=q_{k}^{gb}⊙\left(\frac{T}{2}\omega_{k,gb}^{b}\right)$$

where *T* is the sample period, ⊙ the quaternion product operator and $\omega_{k,gb}^{b}$ the angular velocity, which is directly derived from the gyroscope output:

(10) $$\omega_{k,gb}^{b}=y_{k}^{g}-e_{\omega }$$

where $e_{\omega }$ is an independent and identically distributed (i.i.d.) Gaussian noise source.

Next, using vector measures of the accelerometers, an independent orientation estimate is obtained and can be used to correct for errors caused by integration drift [19].

Prior to the coordinate transformation to the global frame, the force sensor output $y_{f}$ needs to be expressed in the coordinate frame of the local inertial sensors $\Psi_{b}$, which has been done by the knowledge of the finger cuffs’ geometry.

Summarizing, for the force sensor output ($y_{f}^{f}$), the total transformation of the force sensor output is given by:

(11) $$y_{f}^{g}=R\left(q^{gb}\right)R\left(q^{bf}\right)y_{f}^{f}$$

where the orientations are expressed using rotation matrices *R* [27], and the force sensor output is modeled as:

(12) $$y_{f}^{f}=f^{f}+e_{f}^{f}$$

where $e_{f}^{f}$ is modeled as an i.i.d. Gaussian noise source.

Besides orientations, the relative acceleration (${\ddot{p}}_{ti}$), velocity (${\dot{p}}_{ti}$) and position ($p_{ti}$) between the thumb and index finger tips are required as the input for the system identification calculations. This is established using forward kinematics combined with the estimated orientation of different finger and thumb segments, explained in our previous article [22], but with a slight modification because the magnetometer readings are not used to estimate the relative heading. Two different approaches have been used to ensure the observability of the relative orientation during grasp.

1. Global force during manipulations: When an object is grasped with the index finger and thumb and is not displaced by the hand or arm, only a single external force, hence gravity, acts on the object. All other forces should cancel each other out, otherwise the object would be accelerated. This gives the ability to perform an update of the relative orientation update between both tip frames.
  With the assumption that the horizontal components of the average net force in the global frame should be negligible, the following equation holds:
  (13) $$y_{force}=AR^{gh}\left(R^{hi}y_{f}^{i}+R^{ht}y_{f}^{t}\right)+e_{force}$$
  with $y_{f}$ being the force measured on the tip of the thumb and index finger and *R* the rotation matrix describing the relative orientation between either the hand (*h*), thumb tip (*t*), index finger tip (*i*) and global frame (*g*). An i.i.d. Gaussian noise source is indicated with $e_{force}$, and *A* are the directions of the horizontal force component:
  $$A=\left[\begin{array}{ccc} 1 & 0 & 0\\ 0 & 1 & 0 \end{array}\right].$$
2. Relative orientation obtained during movements: Under certain conditions, for instance picking up an object after it has been grasped, it is only the subject’s arm that is moving. That means that all digits are not or only relatively slowly moving with respect to each other. This makes it possible to use the relative accelerations and angular velocities as a measurement update. Both signals are measured in a different sensor frame, but should have the same magnitude. This allows us to update the relative orientation between segments during those pseudo static intervals.
  This can be modeled as:
  (14) $$y_{movement}=R^{12}y_{\left\{g,a\right\}}^{2}-y_{\left\{g,a\right\}}^{1}+e_{movement}$$
  where $R^{12}$ is the orientation between two arbitrary frames and $e_{movement}$ an i.i.d. Gaussian noise term. Hence, this update is only applicable under strict conditions. Testing for these conditions can be done using a test statistics on the measured gyroscope and acceleration signals. Large deviations, i.e., the norm of the difference in measured acceleration or angular velocity signals is not close to zero, are detected using a generalized likelihood ratio test (GLRT) described by Skog et al. [28].

**Figure 4:**
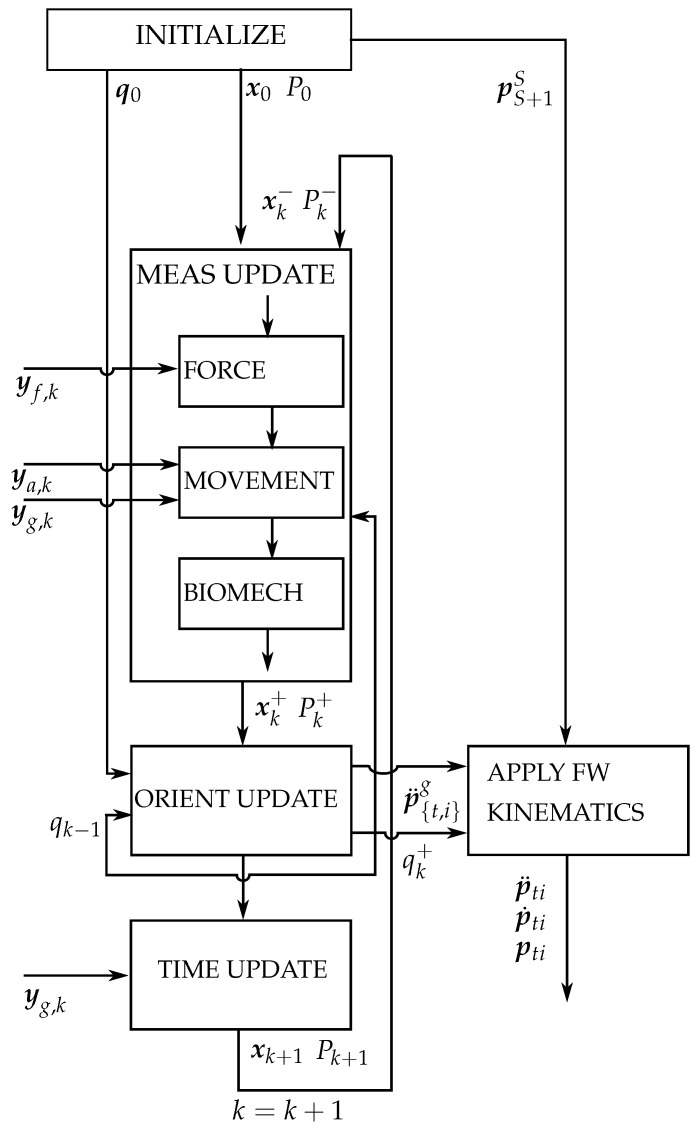
Topology of the kinematic estimation filter embodied in a extended Kalman filter (EKF) that operates on the error states (also knowns a multiplicative extended Kalman filter (MEKF) [26]). The inputs consist of the sensor data ($y_{f}$, $y_{a}$, $y_{g}$) and the lengths of the finger and thumb segments ($p_{S+1}^{S}$). The output consists of the relative kinematics (${\ddot{p}}_{ti}$, ${\dot{p}}_{ti}$, $p_{ti}$) and inertial acceleration of the tips (${\ddot{p}}_{\left\{t,i\right\}}^{g}$). After initialization of both the state (x), error state (δx) and corresponding covariances (*P*), a measurement update of error states according to the measured forces, accelerations, angular velocities ($y_{f}$, $y_{a}$, $y_{g}$) and biomechanical dimensionality information is performed. Information of the force and movement updates can be found in Section 2.2 and Equations (13) and (14). Information of applying biomechanical constraints as a measurement update can be found in our previous article [22]. The orientation states are subsequently used to perform the forward kinematics. Given the set of segmental lengths ($p_{S+1}^{S}$) of the finger and thumb, one can calculate the position, velocity and accelerations of the tips; see also Equation (1) from [22]. Subsequently, gyroscope information $y_{g}$ is used as an input along with a motion model to predict the next state (time update). Finally, the next iteration is initiated (k=k+1), and the described procedure is repeated.

A schematic overview of the implemented kinematic estimation algorithm is given in Figure 4. Hence, this approach is similar as described in our previous research [22] with the difference that: (1) there is no inclusion of magnetometers as their output would have been distorted by the objects that have been grasped; (2) force and gyroscope information is used to update relative orientations during specific phases of the grasp.

### 2.3. Experimental Method

Two different experiments were conducted, each with a different load configuration being handled by the subject.

A subject was seated at a desk with the PowerSensor hardware put on the left hand using Velcro straps; see Figure 1. The forearm and wrist were in a position such that the palm was faced tangential with respect to the table top.

The PowerSensor hardware deploys three triaxial gyroscope (±2000 deg/s) and accelerometer (±4 G) pairs (ST LSM330DLC), one for each finger and thumb segment. In addition, the tips of the index finger and thumb were equipped with the force sensor. Sensor data are sampled (200 Hz for the gyroscope, 100 Hz for the accelerometer, 12 Hz for the force/torque sensors) by a microcontroller (Atmel XMEGA), collected by a master microcontroller (Atmel XMEGA) and subsequently transmitted via USB to the computer. After collecting the sensor data, a sensor to segment calibration was performed as described in our previous article [22]. Then, the kinematics filter and system identification algorithm were executed consecutively. All algorithms are written and executed in customized MATLAB and Python scripts.

In the first experiment, two cylindrical (radius of 3 cm) mass loads (with different mass weights: 0.28 kg and 0.44 kg) were manipulated. The load was initially resting on the table, then grasped by the index finger and thumb, lifted for about 3 s at a height of 0.5 m and eventually placed on the table again. It is assumed that this load corresponds to an infinite stiff internal load, whereas the mass weight parameter is assumed to be finite. There is a change in applied forces on the load as the table exerts a normal force to compensate for the gravitational force at the start and finish of the experiment.

In the second experiment, a passive linear compression spring (spring constant = 0.6 N/mm, zero force length = 4 cm, weight = $10\times 10^{-3}$ kg) was handled. This load corresponds to a finite stiff internal load and a negligible external mass load. The load was handled in a similar way as the mass load; initially resting on the tabletop, picked up and lifted to a height of approximately 20 cm. Now, the spring was repeatedly compressed eight times by the index finger and thumb. Finally, the subject placed the spring load back on the table top. The total force applied by the fingers is assumed to be equal, but opposite (the weight and inertia of the spring are neglected) to the force exerted by the spring. The spring offset position $p_{0}$ was determined at the moment the spring was grasped.

Each experiment was performed by a single subject and repeated 10 times. The medical ethical committee of the Medisch Spectrum Twente (Enschede, NL, The Netherlands) confirmed that no further ethical approval concerning the Medical Research Involving Human Subjects Act (WMO) was required, due to the nature of the study.

## 3. Results

**Figure 5:**
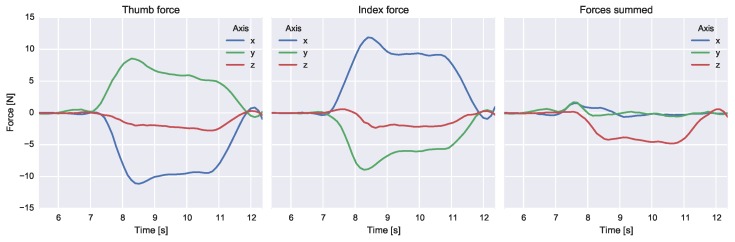
Experiment 1: lifting a mass load (0.44 kg); the forces of the index finger (**left**) and thumb (**middle**). In addition, the summed forces are displayed (**right**). All signals are expressed in the global coordinate frame.

An example reconstruction of the measured 3D forces during the first experiment, i.e., lifting a mass load (0.44 kg), is depicted in Figure 5. Visible are the measured forces starting from grasping the mass load until releasing the load back on the table top. The sum of measured forces expressed in the global reference frame is visible in the right plot. The z-component is primarily determined by the gravitational force exerted on the mass load because inertial accelerations were not dominant compared to the gravitational acceleration acting in the vertical (z) direction.

**Figure 6:**
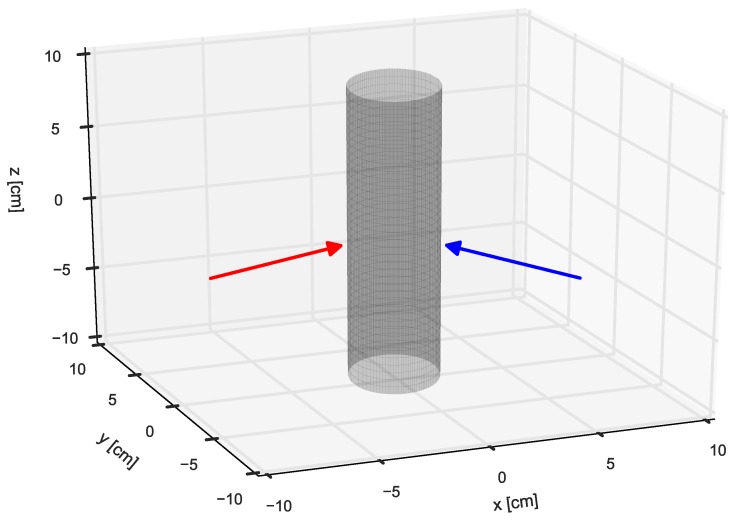
Experiment 1: lifting a cylindrical (radius = 3 cm) mass load (0.44kg); the force vectors for thumb (red) and index finger (blue) are displayed when the load was grasped and held still in the air. Both vectors are expressed in the global reference frame.

A time snapshot (at 11 s) of the same trial is used to reconstruct the measured forces in 3D; see Figure 6. Both measured force vectors are expressed in a global reference frame. It can be seen that both vectors have opposite horizontal and positive vertical components. This indicates that, besides normal forces, also shear forces are present during the grasp.

**Figure 7:**
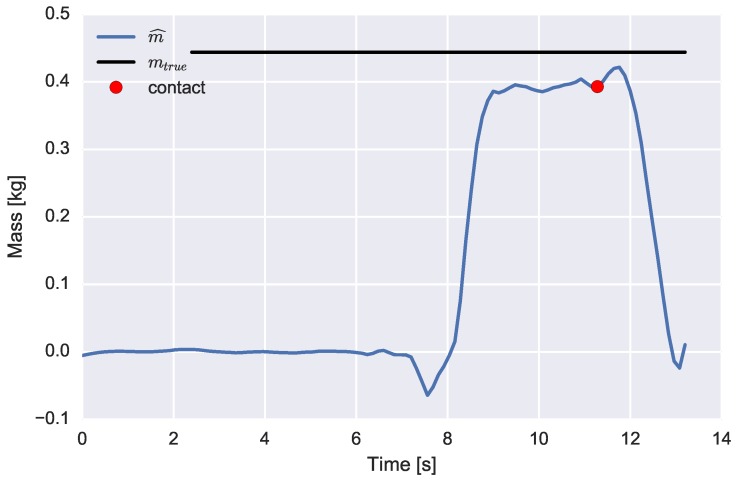
Experiment 1: lifting the cylindrical mass load (0.44kg). The estimated (blue) and true (black) mass weight parameter value are displayed. The load has been grasped at approximately *t* = 7 s. A point estimate of the mass value used for further analysis is indicated by a red dot.

The mass’s weight is estimated online using the RLS algorithm. A point estimate of the estimated weight value is determined at 0.5 s before the release of the object, where it should be noticed that the grasp and release of the object have been determined using a threshold detector acting on the sum of measured forces. An example time series of the, recursively, estimated mass’s weight is depicted in Figure 7.

**Figure 8:**
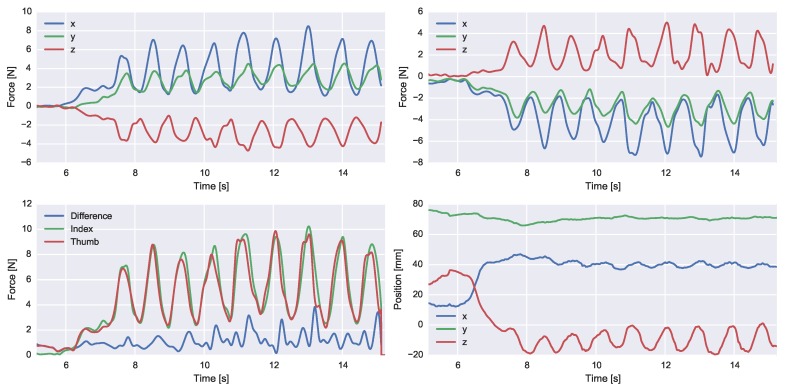
Example reconstruction of Experiment 2 where a spring load was manipulated. A force has been applied to the spring by index finger (**top left**) and thumb (**top right**). The magnitude of both index finger and thumb forces and the magnitude of the sum are depicted in the (**lower left**) graph. The (**lower right**) plot depicts the relative tip position. All signals are expressed in the global coordinate frame.

Figure 8 represents a trial of the second experiment (spring compressions). The 3D forces of the thumb and index finger are depicted, as well as the total force magnitudes and relative distance between both tips. During the experiment, the subject imposed minimal hand accelerations. This has been empirically observed by testing for the difference in the norms of the measured accelerations and the known gravitational acceleration.

**Figure 9:**
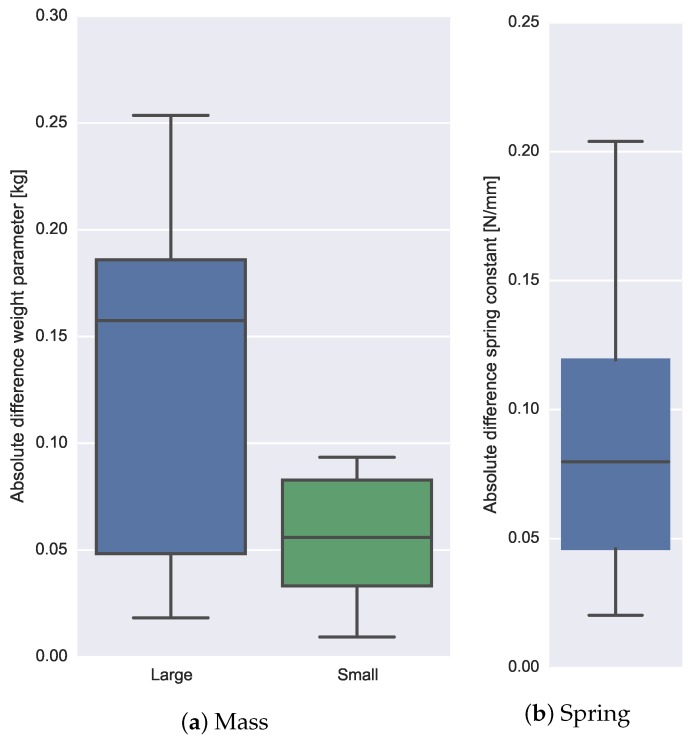
Boxplots of the absolute difference measures of the mass parameter (kg) estimated during Experiment 1 where two different loads were manipulated (**a**). A boxplot of the stiffness parameter (N/mm) estimated during Experiment 2 (**b**) . The median, upper and lower quartiles and outliers (dots) are visualized. All experiments were conducted 10 times.

The estimation error is defined as the absolute difference between the estimated and true value of the mass weight parameter (first experiment) and spring stiffness parameter (second experiment). The experiments were repeated 10 times for both mass weights loads and the spring load. Absolute differences are given in box-plots (see Figure 9a) and were 0.13±0.08 kg (29.3%±18.9%) for the large mass load (0.44 kg) and 0.06±0.03 kg (19.7%±10.6%) for the smaller mass load (0.28 kg).

Like the first experiment, an RLS algorithm was used to estimate the spring stiffness online. A point estimate of the parameter value was, arbitrarily, determined 0.5 s before the release of the spring object. The absolute error values of the estimated spring constant are $8.9\pm 5.7\times 10^{-2}$ (N/mm) (14.8%±9.6%). The variation of differences are visualized in the box-plot; see Figure 9b.

## 4. Discussion

We have demonstrated a novel device that allows the simultaneous measurement of thumb and finger movements and interaction forces with the environment. The system was tested during fairly simple hand motor tasks and was able to reconstruct the important dynamic characteristics of loads being manipulated by the index finger and thumb.

Yet, the proposed device is a prototype and therefore in an experimental status that copes with various, mostly practical, issues. Those issues reflect the quality of the estimated parameters. It is our intention to show the feasibility of such a system. Further sensitivity analyses of different subsystems are required to improve the quality of the output data.

First, attachment of the force sensors to the skin is a major concern. Human tissue deforms easily, especially when it is in contact with external objects. These deformations deteriorate kinematic estimates of motion capture (mocap) systems, often indicated as soft tissue artifacts (STA) [29,30]. STA effects can be reduced if forces induced by movement accelerations are small compared to the forces causing skin deformations. This can be achieved by keeping the sensor lightweight and small.

Second, measuring interaction forces requires a proper coupling between the human tissue, force sensor and the environmental load. Force sensors are inherently stiff, which makes a good skin to sensor attachment rather difficult as the sensor easily tends to shift when forces are applied (STA). In our study, custom-made cuffs were designed and used to attach both force and movement sensors to the tips. The combination of cuff and stiff sensors results in the loss of touch and causes major difficulties in proper handling of the load. In this study, only two force sensors were used. Hence, grasping an object forces the subject to pinch between the index and thumb finger. If the center of mass (CoM) of the object is not positioned between the application points, an undesired moment is applied around the force sensor’s normal sensitive axis. Stability could be improved significantly when three or more fingers are used for grasping.

Third, the electronic data acquisition circuitry requires improvements as it is currently sensitive for power supply fluctuations, which especially affect the daisy-chained force sensors. All force sensor data have been low pass filtered prior to further processing in order to minimize this effect as much as possible. Further improvements can be made in the sampling rate of the force sensor, which has been capped as the sensor used a 100-Hz clock to read each of the 12 force channels serially. The effective sample frequency is restricted to 8.3 Hz, whereas the individual channels should preferably be sampled at a much higher rate (>100 Hz). In our study, a cubic interpolator was used in order to feed the Kalman filter with force updates at a sufficient rate.

Fourth, as mentioned in the Methods section, it is necessary to express both force sensors in a common coordinate frame. The inertial sensors were used for this purpose. Prior to using the inertial sensors for kinematics estimates, a mapping (sensor to segment calibration) is required. Obtaining a proper calibration is a bit difficult because the subject is constrained in his/her movements due to the apparatus being attached to the subject’s hand. Another drawback of the inertial sensors is that the gyroscope and accelerometer pair provide only the relative orientation during sufficient common movement. In the static situation, only the relative inclination is provided. A traditional inertial and magnetic measurement unit (IMMU) provides heading information using a magnetometer. However, large disturbances due to the vicinity of magnetic materials make this approach challenging. In addition, the relative orientation between the rigid force and inertial sensor should be known. An improved version of the kinematic estimator is currently under development, which should avoid the usage of the magnetometers, but still provides robust heading estimates.

Any of the mentioned errors could have been accumulated, which resulted in a fairly large mismatch between the true and estimated parameter values.

Two different loads, indicated as a mass and a spring, were manipulated. They do not only differ in dynamic characteristics, but also in the way they are handled; the mass was primarily perturbed by an external gravitational force, whereas the spring was perturbed by internally-applied finger forces. Those two distinct load types are common in ADL tasks where grasping and releasing a load involves gripping forces and displacing a load involves one or more external force sources. The way forces act differently on both loads could also be an explanation for the relatively large errors of the spring’s constant estimate compared to the mass’ parameter estimates. The estimation quality of the spring relies heavily on the relative kinematics, whereas the mass estimate only requires a good estimate of the inclination during static, that is minimal movement, periods. The estimate of the relative orientation between the thumb an index finger tips could be improved when heading information is available during the complete movement and not only during the limited periods of the contact.

In our study, the external dynamics were assumed to be rather simple, as only the gravitational acceleration was dominant. Higher order dynamics could be implemented fairly easily for various loads, like drag frictions and inertial accelerations. The latter would be practically easy, but requires either a fairly large mass or a large force sensor bandwidth, which are both troublesome for the current setup.

Independent perturbations are required to identify parts of the closed loop system. Depending on the type (movement or force) and location where the perturbation enters the loop, different parts of the loop will be identified. Further research is required to analyze the estimation validity using measures of interaction forces and movement under such various conditions and excitations.

A possible application in the rehabilitation field of the proposed device is the classification and quantification of various hand motor tasks. Especially in neurological diseases, it is often the case that the disease resulted in spasticity of the arm and hand muscles. Not only the grasps (e.g., power grip, pinching, span) could be assessed, but also the planning of the tasks and, hence, the strategy of different subjects aimed at performing a single task could be monitored using our device. Quantitative measures of not only the forces applied to an environmental object, but also measures from the corresponding arm and hand movements give much more information (e.g., power exchange) about the efficiency by which the subject is performing the specific task.

Further application areas can be found in machine learning, like the classification of various handled objects. Obviously, a physical interpretation of the quantitative outcomes might be impossible, but the requirements of the sensing hardware could be less strict.

The current hardware has a large advantage, as it measures in multiple directions and, therefore, might enable the estimation of complex load dynamics, like a mug with a viscous or granular content.

## 5. Conclusions

Body-worn unobtrusive sensing systems are of utmost importance for interaction measurements in ADL tasks.

The development and preliminary validation of a hand-worn 3D force and movement setup has been presented. The setup allows for measurements of independent finger movements and forces using a custom-made hardware that includes 3D MEMS-based force and inertial sensors, which can be worn on small body surfaces, like finger tips.

Two loads were manipulated by the hand, and their dynamic characteristics were estimated by simultaneously measuring kinematic and kinetic sensor data, applying orientation filters and subsequently applying a recursive system identification algorithm.

The systems goes beyond pressure sensing and gesture recognition and, therefore, opens the ability to gather profound insights into interaction optimization between the human body and its environment.

## Figures and Tables

**Figure A1:**
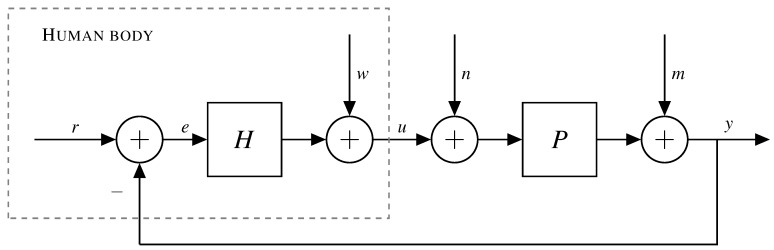
Closed loop representation of human (*H*) load (*P*) interaction. The human body (dashed rectangle) is modeled as a setpoint controller, dynamics (*H*) and an independent active force generator (*w*). Independent force (*n*) and position disturbances (*m*) are given.

## Appendix A. Closed Loop System Identification

Figure A1 models the closed loop coupling between the human body (dashed rectangle) and environmental load (*P*). The human body (dashed box) consists of a set-point controller with reference position signal (*r*), dynamics (*H*) and a independent force source (*w*). External force (*n*) and position disturbances (*m*) and both inputs are assumed independent. Hence, this can be shown in the frequency domain with the cross-spectral densities being zero:

(A1) $$S_{rn}=S_{rw}=S_{rm}=\cdots =0.$$

We measure both positions *y* and forces *u*. Now, the frequency response function (FRF) of the load is given by:

(A2) $$\hat{P}=\frac{S_{uy}}{S_{uu}}$$

The Fourier transforms of *u* and *y* are given by:

(A3) $$\begin{array}{cc} U(\omega )= & SHR+SW-SHM-SHPN \end{array}$$

(A4) $$\begin{array}{cc} Y(\omega )= & SHPR+SPW+SPN+SM \end{array}$$

where the sensitivity *S* is given by:

(A5) $$S\left(\omega \right)=\frac{1}{1+H(\omega )P(\omega )}$$

Now, the cross- and auto-spectral density are given by:

(A6) $$\begin{array}{cc} S_{uy} & =\frac{1}{T}U^{*}Y\\ & =\frac{{\left|S\right|}^{2}}{T}\left({\left|H_{0}\right|}^{2}PS_{rr}+PS_{ww}-H^{*}S_{mn}-H^{*}{\left|P_{0}\right|}^{2}S_{nn}\right) \end{array}$$

(A7) $$\begin{array}{cc} S_{uu} & =\frac{1}{T}U^{*}U\\ & =\frac{{\left|S\right|}^{2}}{T}\left({\left|H_{0}\right|}^{2}S_{rr}+S_{ww}-{\left|H_{0}\right|}^{2}S_{mn}-{\left|H_{0}\right|}^{2}{\left|P_{0}\right|}^{2}S_{nn}\right) \end{array}$$

where the asterisk symbol indicates the complex conjugate and:

(A8) $$S^{*}S={\left|S\right|}^{2}$$

Finally, one yields the FRF estimate of the plant [31,32]:

(A9) $$\begin{array}{cc} \hat{P} & =\frac{S_{uy}}{S_{uu}}\\ & =\frac{{\left|H_{0}\right|}^{2}PS_{rr}+PS_{ww}-H^{*}S_{mm}-H^{*}{\left|P_{0}\right|}^{2}S_{nn}}{{\left|H_{0}\right|}^{2}S_{rr}+S_{ww}-{\left|H_{0}\right|}^{2}S_{mm}-{\left|H_{0}\right|}^{2}{\left|P_{0}\right|}^{2}S_{nn}} \end{array}$$

If $S_{ww}$ dominates w.r.t. other noise sources, $\hat{P}$ approximates *P*. However, if any other noise source dominates, the estimate $\hat{P}$ approximates $-\frac{1}{H}$.
